# All-optical tunable wavelength conversion in opaque nonlinear nanostructures

**DOI:** 10.1515/nanoph-2022-0078

**Published:** 2022-05-30

**Authors:** Jiannan Gao, Maria Antonietta Vincenti, Jesse Frantz, Anthony Clabeau, Xingdu Qiao, Liang Feng, Michael Scalora, Natalia M. Litchinitser

**Affiliations:** Department of Electrical and Computer Engineering, Duke University, Durham, NC, 27708, USA; Department of Information Engineering, University of Brescia, Via Branze 38, 25123 Brescia, Italy; US Naval Research Laboratory, 4555 Overlook Ave., SW, Washington, DC 20375, USA; University Research Foundation, 6411 Ivy Ln. 110, Greenbelt, MD 20770, USA; Department of Electrical and Systems Engineering, University of Pennsylvania, Philadelphia, PA 19104, USA; Department of Materials Science and Engineering, University of Pennsylvania, Philadelphia, PA 19104, USA; Aviation and Missile Center, US Army CCDC, Redstone Arsenal, Huntsville, AL 35898-5000, USA

**Keywords:** all-optical tunabilitiy, chalcogenide, femtosecond optics, nonlinear metasurface, phase-locking

## Abstract

We demonstrate a simple, femtosecond-scale wavelength tunable, subwavelength-thick nanostructure that performs efficient wavelength conversion from the infrared to the ultraviolet. The output wavelength can be tuned by varying the input power of the infrared pump beam and/or relative delay of the control beam with respect to the pump beam, and does not require any external realignment of the system. The nanostructure is made of chalcogenide glass that possesses strong Kerr nonlinearity and high linear refractive index, leading to strong field enhancement at Mie resonances. Although, as many other materials, chalcogenide glasses absorb in the ultraviolet range, fundamental phase-locking mechanism between the pump and the inhomogeneous portion of the third-harmonic signal enables ultraviolet transmission with little or no absorption.

## 1 Introduction

Coherent ultraviolet (UV) sources are essential for a number of science and technology applications, including flow cytometry, sensing, data storage, semiconductor processing, and military and space applications [1–7]. Coherent UV light is mostly produced using frequency conversion in nonlinear optical crystals, as in a frequency-doubling optical parametric amplifier pumped by a femtosecond amplified Ti:sapphire laser. Although versatile, such systems are usually bulky, and UV generation on a chip remains challenging. Conventional wisdom currently dictates that the practical realization of an integrated, compact, tunable UV source requires a nonlinear optical material that is transparent in the UV, and possesses high and fast nonlinear susceptibilities and birefringence, thus enabling phase-matching and efficient wavelength conversion. The quest to find a material that satisfies all these criteria simultaneously is not trivial. The emergence of nonlinear optical metasurfaces opened an entirely new avenue in the broad field of wavelength conversion [8–13].

One of the most commonly used materials for nonlinear optical metasurfaces is silicon (Si). Si-based nonlinear metasurfaces have been demonstrated for both the enhancement and tunability of third harmonic generation (THG) based on the generation of free carriers in highly doped environments. In reference [14], it was shown that the refractive index of a Si metasurface changes because of photo-induced generation of free carriers (FCs) in the mid-infrared. Similarly, frequency conversion due to the photo-induced time-variant refractive index in the near-infrared spectral range has been reported in germanium-based metasurface [15]. We note, however, that the dynamical aspects of free-carrier generation are different, require different theoretical assumptions and approaches, and the speed of the free-carrier generation-based tunability in usually on the picosecond or longer time scale [16]. Moreover, it is much slower than the nonlinear Kerr effect, significantly limiting its applications in ultrafast optics. In contrast, epsilon-near-zero (ENZ) assisted high-harmonic generation resulting from the photo-induced electron heating and the consequent time-dependent ENZ wavelength was reported in reference [17]. However, ENZ wavelength tuning is limited by the degree of doping of the constituent materials [18], and post-deposition annealing. These characteristics once again display a different electrodynamic response, require a different set of assumptions, and limit the performance of such a wavelength converter to a narrow wavelength range near the ENZ crossing point.

Recently, we demonstrated near-infrared to ultraviolet frequency conversion in a chalcogenide glass (ChG) metasurface, enabled by a phase-locking mechanism between the pump and the inhomogeneous portion of the third harmonic (TH) signal [19]. Phase locking [20–27] allows the co-propagation of the pump pulse and the inhomogeneous harmonic component, which acquires the same refractive index and absorption coefficient as the infrared pump. When this process occurs in a cavity, efficient frequency conversion occurs despite the presence of strong material absorption at the harmonics’ wavelengths [21, 28, 29]. ChGs, such as As_2_S_3_ or As_2_Se_3_, possess strong and fast cubic nonlinearities with a femtosecond response time [30]. Expanding their use from traditional near- and mid-infrared portions of the electromagnetic spectrum to the UV range is likely to unlock unprecedented opportunities for developing new, compact, ultrafast tunable sources of coherent UV radiation that can be integrated on existing semiconductor platforms.

## 2 Results and discussion

**Figure 1:**
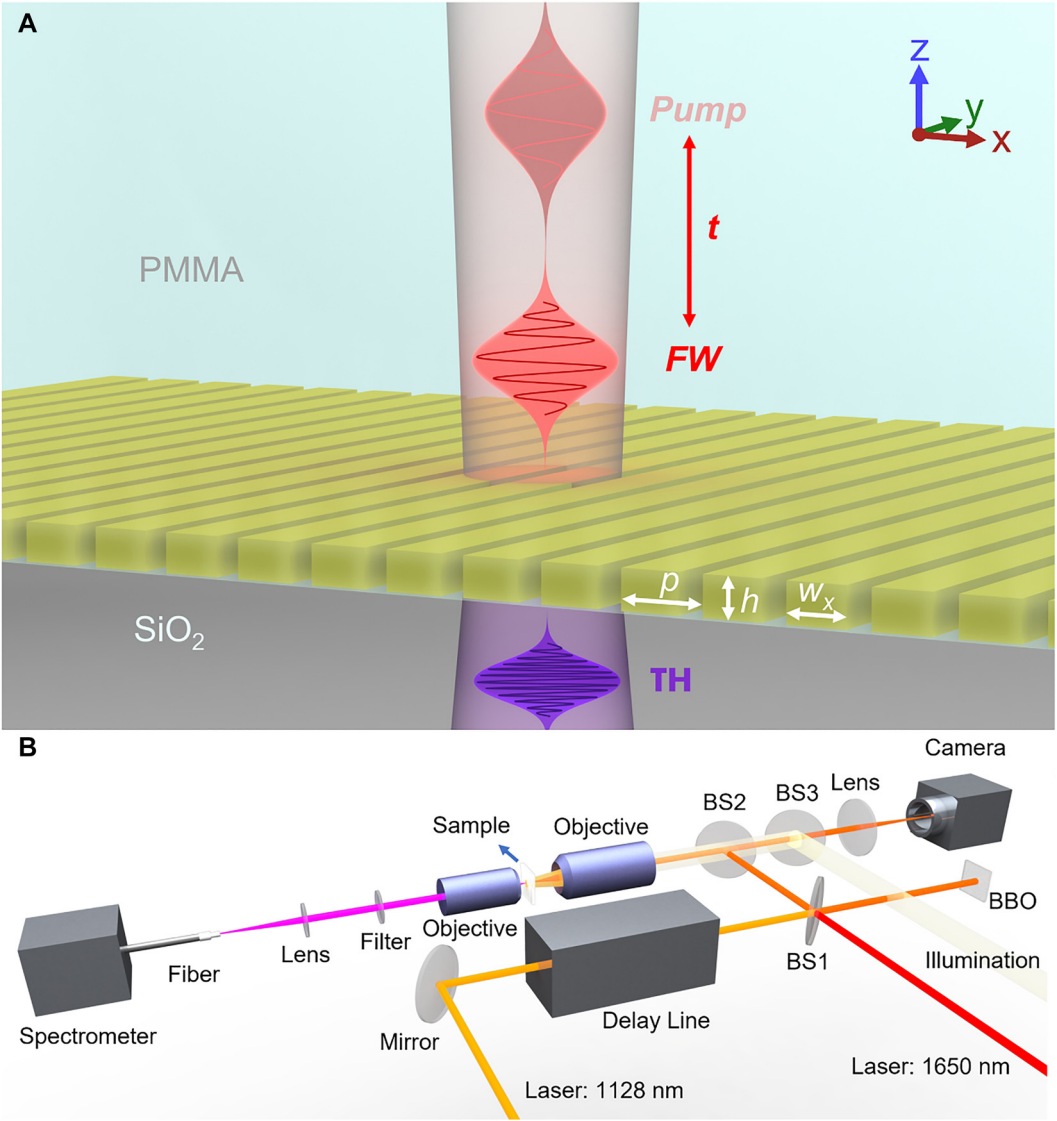
Tunable third harmonic generation in a chalcogenide metasurface. (A) Arsenic trisulfide (As_2_S_3_) metasurface on a fused silica substrate, covered by the PMMA. The area of As_2_S_3_ patterned with nanowires is 400 × 400 μm with a period *p* = 625 nm, nanowire width *w*_x_ = 442 nm, and height *h* = 300 nm. (B) The diagram of the experimental setup that can be used in a single pump (1128 nm pump “on”, 1650 nm “control” pump “off”), or a double-pump configuration (both 1128 and 1650 nm pumps on). In a single pump configuration, the sample is illuminated by near-infrared 100 fs pulses emanating from a tunable ultrafast laser system consisting of a 1 kHz Ti:sapphire laser and optical parametric amplifiers (OPA), polarized along the *x* direction to generate a TH. In a double-pump configuration, a “control”, off-resonance pump at 1650 nm is employed to optically tune the refractive index of As_2_S_3_ based on kerr effects.

Here, for the first time to our knowledge, we experimentally demonstrate femtosecond-speed wavelength tuning of the TH signal generated in a nonlinear ChG metasurface at UV wavelengths, despite the presence of strong material absorption in this range. The metasurface consists of As_2_S_3_ nanowires patterned on a glass substrate, followed by spin coating polymethyl methacrylate (PMMA) on top (see Figure 1A) so that the metasurface is surrounded by materials having the same refractive index, which has a two-fold outcome: significantly improved field localization inside the As_2_S_3_ nanowires compared with the structure without PMMA (air on top), and protecting the As_2_S_3_ structure from degeneration. The geometrical parameters of the metasurface were optimized to enhance THG efficiency in the UV wavelength range by a Mie resonance located at 1119 nm. The period of the nanostructure is *p* = 625 nm, and the width and height of the nanowires are *w*_x_ = 442 nm and *h* = 300 nm, respectively. The electric field of the incident beam at the fundamental frequency (FF) is polarized along the *x* direction. The refractive index of a 300 nm-thick As_2_S_3_ film is measured using a spectroscopic ellipsometer (see Supplementary Section 1) and was used in our design and numerical simulations. The data indicate that As_2_S_3_ is highly dispersive and strongly absorptive at wavelengths below 500 nm, where our third harmonic wavelength (376 nm) is generated. We illuminated the sample with a 100 fs pump pulse having a carrier wavelength of 1128 nm. We recorded the TH signal near 376 nm using a spectrometer. We then explored the tunability of the TH signal by the following two mechanisms. First, we study the power-dependent tunability of the device. By increasing the intensity of the fundamental beam from 956 MW/cm^2^ to 2.87 GW/cm^2^, the observed redshift of the TH signal that is directly attributable to the refractive index change of As_2_S_3_ as a function of the input beam intensity. Next, we further explored the response time of the THG shift by introducing a second, ultrashort laser pulse having carrier wavelength centered at 1650 nm (away from any Mie resonance of the nanostructure, see Supplementary Section 2) and intensity of 36.6 GW/cm^2^ as a control beam. We thus demonstrated the possibility of controlling the shift by adjusting the delay between the pump and control beam. The presence of the control beam modifies the refractive index due to the Kerr nonlinearity, such that *n* = *n*_*l*_ + Δ*n*, where Δ*n* = *n*_2_ *I*, *n*_*l*_ and *n*_2_ are the linear refractive index and the nonlinear coefficient of As_2_S_3_, respectively, and *I* is the intensity of the control beam. When the two incident pulses are synchronized both in time and position on the metasurface, a frequency shift of the FF and TH is maximized.

The metasurface was nanofabricated using standard electron-beam lithography (EBL) and inductively coupled plasma etching (ICP) procedures, followed by spin coating of a PMMA layer on top of the nanostructure (see Section 4).

**Figure 2:**
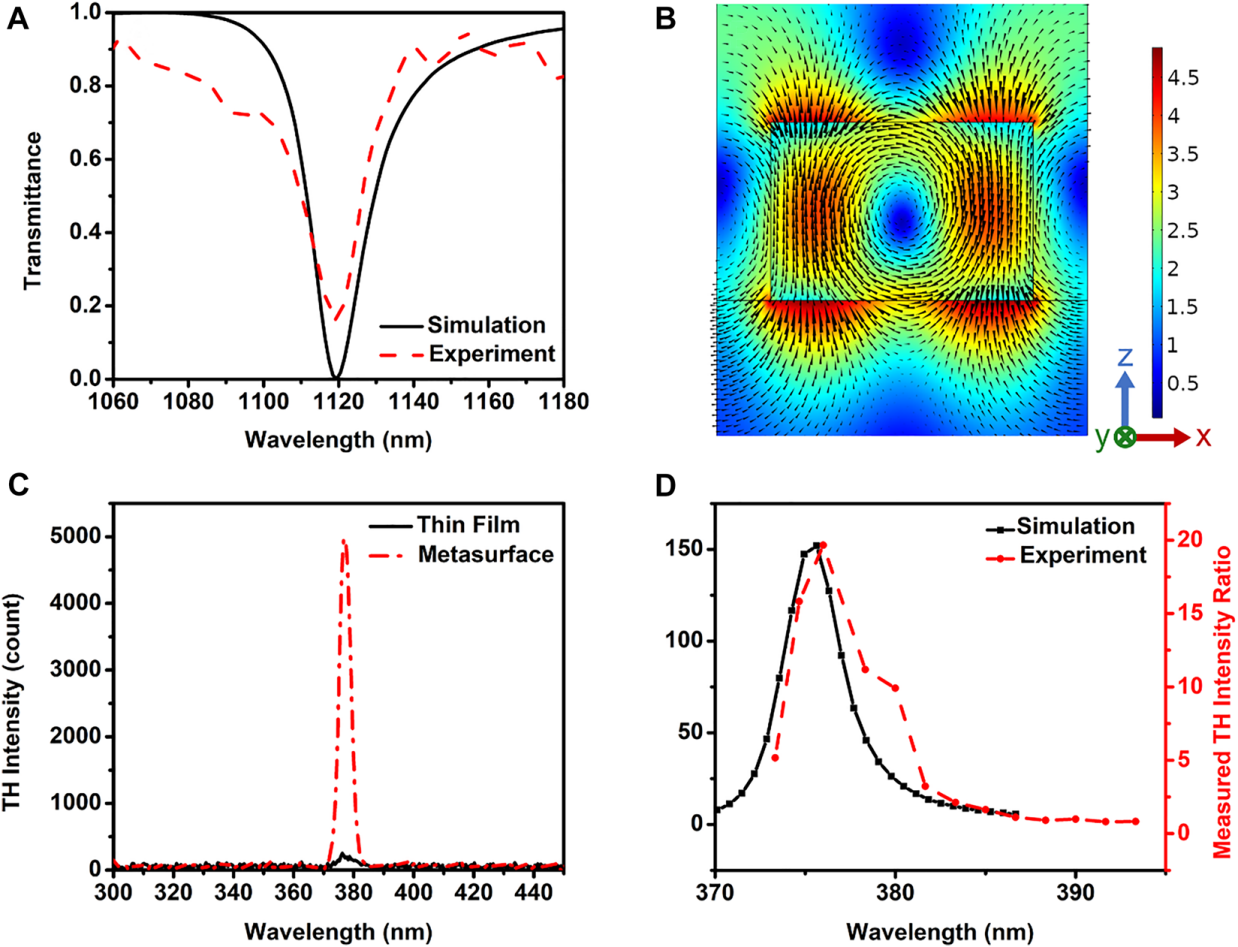
The transmittance and THG from the As_2_S_3_ metasurface. (A) Simulated (black, solid curve) and measured (red, dashed curve) transmittance of the designed sample. The transmission dip appearing at around 1119 nm agrees between numerically simulated transmittance and experimental results. (B) Electric field enhancement ratio between the metasurface sample with *w*_x_ = 442 nm, and the incident electric field at 1128 nm, which is close to the resonance dip at 1119 nm. The arrows showing the rotating electric field indicate the formation of the magnetic-dipole-like Mie resonance. (C) TH intensity measured for the uniform thin film of As_2_S_3_ covered by PMMA (black, solid curve) and for the nanowire-based metasurface (red, dashed-dotted curve). The peak intensity of the THG from metasurface is about 20 times larger than that from the reference sample. (D) The simulated (black, solid curve) and experimental (red, dashed curve) results of the THG enhancement for the metasurface as compared to that of the reference sample.

Figure 2A shows the simulated and measured transmittances of the sample, at wavelengths ranging from 1060 to 1180 nm. The magnetic field displays a minimum at the Mie resonance at 1119 nm (not shown in the figure), corresponding to the position of the minimum in transmittance. Figure 2B shows that the electric field inside the nanowire is enhanced approximately 4.5 times compared to the incident electric field when the laser is tuned to 1128 nm, leading to the enhancement of THG. We stress that the maximum enhancement for the electric field occurs at 1128 nm, and is slightly detuned from the Mie resonance peak of the nanostructure and where THG peaks. At low intensity, the linear Mie resonance peaks at 1119 nm. An incident peak intensity of 956 MW/cm^2^ shifts the resonance by approximately 3–4 nm, a change triggered by the *χ* ^(3)^ of the material.

In order to measure the TH signal, the sample was illuminated with the FF pump beam centered at a wavelength of 1128 nm, with beam waist of 35 µm, and peak intensity of 956 MW/cm^2^. The intensity is significantly lower than the surface damage threshold for As_2_S_3_ glass [31]. In Figure 2C we compare the peak TH intensities generated from the metasurface and the unpatterned reference sample. The peak TH signal measured from the metasurface is about 20 times larger than the TH signal emanating from the reference sample. As expected, this maximum contrast between the two signals occurs near the Mie resonance, at 1128 nm, in good agreement with the calculated results, as shown in Figure 2D. We note that the experimentally measured intensity of the generated TH is smaller than the predicted intensity. Also, the TH is generated at a slightly longer wavelength compared to the location of the predicted peak. We consider the following possible reasons that could lead to these discrepancies: (i) nanofabrication related non-uniformity in nanowire dimensions and periodicity, which could result in broadening of the width of the resonance and a decreased THG efficiency; (ii) the laser beam at FF has a finite angular distribution of wavevectors after the objective, resulting in an effective incident angle slightly off of the normal, which we assumed in the numerical simulations. (iii) The effective value of *χ* ^(3)^ in the As_2_S_3_ material system may be higher than the theoretically predicted value, therefore causing a somewhat larger shift of the Mie resonance when illuminated with the laser beam.

**Figure 3:**
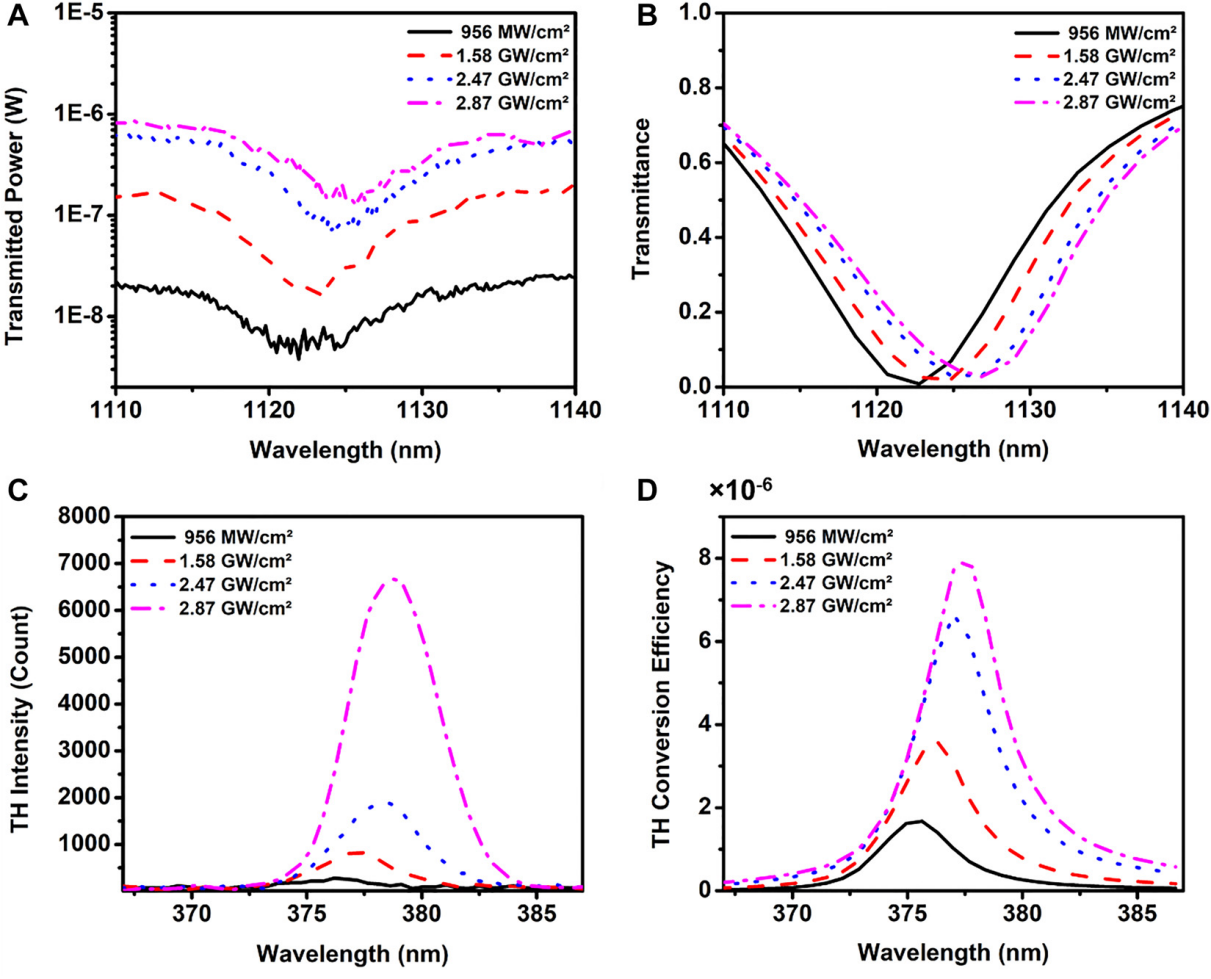
Measurement results of power-dependent Mie resonance position and THG. The experimental (A) and simulated (B) results of red shift of the Mie resonance dip with the increase of the intensity of the fundamental beam. The linear transmittance dip in Figure 2A is at 1119 nm and moves to 1122 nm when the pump laser with 956 MW/cm^2^ is applied. After increasing the pump beam intensity further to 2.89 GW/cm^2^, the dip moves to 1126 nm. The transmitted power is measured by the pumping at wavelength of 1128 nm with the femtosecond laser. The measured (C) and simulated (D) shift of THG with the increase of the intensity of the fundamental beam. The THG efficiency is calculated by the THG power divided by the fundamental beam power.

Next, we study the power-dependent tunability of the device. Figure 3A shows that increasing the intensity of the FF beam from 956 MW/cm^2^ to 2.87 GW/cm^2^ yields a 7 nm redshift of the transmission minimum relative to that shown in Figure 2A (also shown by the black curve in Figure 3A). The curves in Figure 3B are the results of numerical simulations that also demonstrate a redshift of the transmission spectrum minimum, in good agreement with the experimental data. Figure 3C and D shows the corresponding measured and simulated 2.5 nm redshift of the THG. These shifts may be directly attributed to the refractive index change of As_2_S_3_ as a function of the input beam intensity. We note that the measured shift is slightly larger compared to that in numerical simulations, probably due to the fact that the value of *χ* ^(3)^ in our As_2_S_3_ material system is slightly larger than the predicted value using our theoretical model [32]. Nevertheless, good qualitative and quantitative agreement between data and simulations is evident.

The Kerr response of chalcogenides is one of the fastest known responses compared with other tunability mechanisms such as phase-change, free carrier, thermal effects [33]. Since the wavelength of the pump laser we employed in the experiments is always set inside the finite bandwidth of the Mie resonance, the response time should be limited only by the duration of the laser pulse we used in the measurements. In order to explore the response time of this THG shift, a pump-probe experiment was designed based on the following theoretical consideration. In the second scenario, the sample is illuminated by two pump beams at two separate frequencies *ω*_1_ and *ω*_2_: *ω*_1_ is a pump beam used for generating TH and *ω*_2_ is a control beam used for changing the refractive index of the material induced by Kerr effects. The expression for two pumps and two polarization components at two separate frequencies is:

(1) $$P_{bj}^{3}={\left(P_{y}^{\omega_{1}}{\text{e}}^{-\text{i}\omega_{1}t}+{\left(P_{y}^{\omega_{1}}\right)}^{\text{*}}{\text{e}}^{\text{i}\omega_{1}t}+P_{y}^{\omega_{2}}{\text{e}}^{-\text{i}\omega_{2}t}+{\left(P_{y}^{\omega_{2}}\right)}^{\text{*}}{\text{e}}^{\text{i}\omega_{2}t}\right)}^{3},$$

where *P*_*bj*_ is the total polarization component, *P*_*y*_^*ω*1^ is the polarization component from pump at frequency *ω*_1_ and *P*_*y*_^*ω*2^ is the polarization component from control beam at frequency *ω*_2_. After expanding the righthand side of Eq. (1) and dropping terms that oscillate at sum and difference frequencies, we obtain two terms that oscillate at the pump and control beam frequencies:

$$\begin{array}{ll} {P_{bj}}^{3} & =\left(\begin{array}{c} {\left(P_{y}^{\omega_{1}}\right)}^{2}{\text{e}}^{-\text{i}2\omega_{1}t}+{\left(P_{y}^{\omega_{1}}\right)}^{\text{*}2}{\text{e}}^{\text{i}2\omega_{1}t}+2P_{y}^{\omega_{1}}{\left(P_{y}^{\omega_{1}}\right)}^{\text{*}}\\ +{\left(P_{y}^{\omega_{2}}\right)}^{2}{\text{e}}^{-\text{i}2\omega_{2}t}+{\left(P_{y}^{\omega_{2}}\right)}^{\text{*}2}{\text{e}}^{\text{i}2\omega_{2}t}+2P_{y}^{\omega_{2}}{\left(P_{y}^{\omega_{2}}\right)}^{\text{*}}\\ \left(2P_{y}^{\omega_{1}}P_{y}^{\omega_{2}}{\text{e}}^{-\text{i}\omega_{2}t}{\text{e}}^{-\text{i}\omega_{1}t}+2{\left(P_{y}^{\omega_{1}}\right)}^{\text{*}}P_{y}^{\omega_{2}}{\text{e}}^{-\text{i}\omega_{2}t}{\text{e}}^{\text{i}\omega_{1}t}\right)\\ \left(2P_{y}^{\omega_{1}}{\left(P_{y}^{\omega_{2}}\right)}^{\text{*}}{\text{e}}^{\text{i}\omega_{2}t}{\text{e}}^{-\text{i}\omega_{1}t}+2{\left(P_{y}^{\omega_{1}}\right)}^{\text{*}}{\left(P_{y}^{\omega_{2}}\right)}^{\text{*}}{\text{e}}^{\text{i}\omega_{2}t}{\text{e}}^{\text{i}\omega_{1}t}\right) \end{array}\right)\\ & \;\cdot \left(P_{y}^{\omega_{1}}{\text{e}}^{-\text{i}\omega_{1}t}+{\left(P_{y}^{\omega_{1}}\right)}^{*}{\text{e}}^{\text{i}\omega_{1}t}+P_{y}^{\omega_{2}}{\text{e}}^{-\text{i}\omega_{2}t}+{\left(P_{y}^{\omega_{2}}\right)}^{*}{\text{e}}^{\text{i}\omega_{2}t}\right) \end{array}$$

(2) $$\begin{array}{ll} {P_{bj}}^{3} & \approx \left[\left(2P_{y}^{\omega_{1}}{\left(P_{y}^{\omega_{1}}\right)}^{\text{*}}+2P_{y}^{\omega_{2}}{\left(P_{y}^{\omega_{2}}\right)}^{\text{*}}\right)P_{y}^{\omega_{1}}+2P_{y}^{\omega_{1}}P_{y}^{\omega_{2}}{\left(P_{y}^{\omega_{2}}\right)}^{\text{*}},+2P_{y}^{\omega_{1}}{\left(P_{y}^{\omega_{2}}\right)}^{\text{*}}P_{y}^{\omega_{2}}\right]{\text{e}}^{-\text{i}\omega_{1}t}\\ & \;+\left[\left(2P_{y}^{\omega_{1}}{\left(P_{y}^{\omega_{1}}\right)}^{\text{*}}+2P_{y}^{\omega_{2}}{\left(P_{y}^{\omega_{2}}\right)}^{\text{*}}\right)P_{y}^{\omega_{2}}+2P_{y}^{\omega_{1}}P_{y}^{\omega_{2}}{\left(P_{y}^{\omega_{1}}\right)}^{\text{*}},+2{\left(P_{y}^{\omega_{1}}\right)}^{\text{*}}P_{y}^{\omega_{2}}P_{y}^{\omega_{1}}\right]{\text{e}}^{-\text{i}\omega_{2}t}. \end{array}$$

If *P*_*y*_^*ω*_2_^>>*P*_*y*_^*ω*_1_^, then Eq. (2) reduces to:

(3) $$P_{bj}^{3}\approx 6|P_{y}^{\omega_{2}}{|}^{2}P_{y}^{\omega_{1}}{\text{e}}^{-\text{i}\omega_{1}t}+2|P_{y}^{\omega_{2}}{|}^{2}P_{y}^{\omega_{2}}{\text{e}}^{-\text{i}\omega_{2}t}.$$

**Figure 4:**
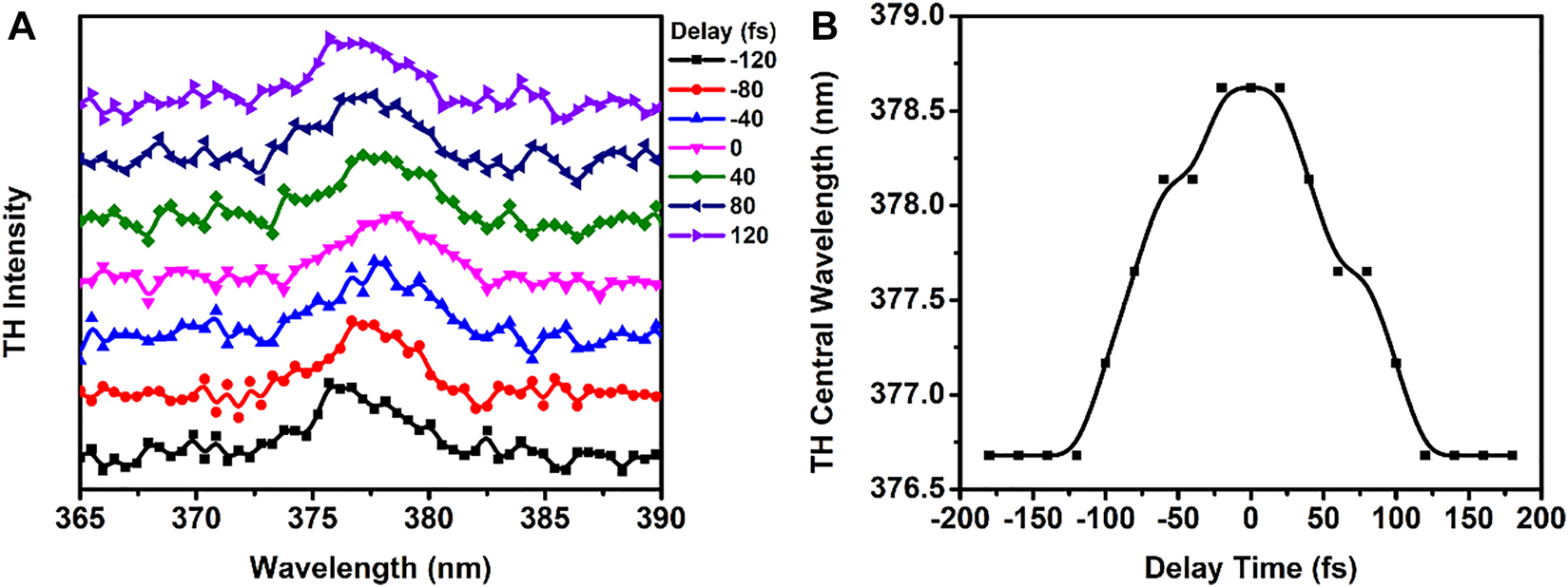
The pump-probe experimental results of THG. (A) The peak of the collected TH shifts with the delay time. (B) The central wavelength of the TH as a function of the delay time.

Equation (3) shows that the main driving third order term for the weak pump at *ω*_1_ is the strong control beam at *ω*_2_, leading to the Kerr-effect-based frequency tuning of the pump at *ω*_1_. Tunability of the weak pump at *ω*_1_ results into the tunability of the TH frequency as shown in Figure 4.

The experiment was then performed, and the diagram of the pump-probe setup is shown in Figure 1B. The output of the Ti:sapphire laser with a central wavelength of 800 nm, repetition rate of 1 kHz, and pulse duration of 100 fs was split and routed into two separate OPAs that converted it to the near-infrared wavelengths of 1128 and 1650 nm. The pulses at these new wavelengths were recombined at the beam splitter 1. A mechanical delay line system positioned along the propagation path of the 1128 nm laser beam was used to control the delay time between the two pulses. After the beam splitter, one branch of the two beams is focused onto the sample by the infinity-corrected objective with an NA of 0.5. The other branch was transmitted through the beta barium borate (BBO) crystal to determine the synchronization status of the two pulses. A 4f system consisting of the objective and an achromatic lens was used for sample imaging and alignment. Another 4f system was setup to collect the TH signal generated by the sample, and couple the TH signal into a spectrometer through a multimode fiber (400 µm core, 0.5 NA). The intensities of the 1128 nm and 1650 nm beams were 956 MW/cm^2^ and 36.6 GW/cm^2^, respectively. The 30°-tilted scanning electron microscopy (SEM) image of the As_2_S_3_ pattern is shown in Supplementary Section 3.

Figure 4A shows that the TH peak is moving back and forth when the delay time changes from −120 fs to 120 fs. We then plot the central TH wavelength versus delay time (Figure 4B) and find that the duration of the TH shift is about 200 fs, twice as long as pump pulse duration. These results clearly demonstrate that this all-optical tunability occurs on the femtosecond timescale and that the response speed is limited only by pulse duration. This is in agreement with our predictions based on the unique ultrafast property of Kerr effect. The peak intensity of the pump beam at 1650 nm can be increased to further increase the range of the tunability. It is likely that the maximum peak pump intensity can be increased by replacing As_2_S_3_ with a ChG having a composition optimized to maximize the threshold for the onset of damage (e.g., Ge_20_As_20_Se_60_) [34], and by passivating the surface with a thin layer of alumina [35].

## 3 Conclusions

In summary, we theoretically predicted and experimentally demonstrated the ultrafast tunability of THG generated in the opaque spectral range of As_2_S_3_ glass metasurfaces. The THG is enabled by a phase-locking mechanism and enhanced by the field localization at the Mie resonance of the nanostructure. The femtosecond-speed tunability is facilitated by the large Kerr nonlinearity of the As_2_S_3_. These results could greatly extend our ability to control nonlinear light–matter interactions as the phase locking mechanism observed here can be applied to other nonlinear material systems [36] and may be useful for the development of on-chip ultrafast switches, all optically controlled nano-lasers and other flat optics-based nonlinear nanophotonic devices.

## 4 Methods

### 4.1 Samples preparation

The As_2_S_3_ film with a thickness of 300 nm was deposited on top of a glass substrate via thermal deposition in a Lesker PVD 75 deposition system equipped with a low temperature evaporation source. The substrate temperature was controlled at approximately 20 °C during the deposition. The linear refractive index of the deposited As_2_S_3_ thin film was measured using a VASE, J.A.Woollam spectroscopic ellipsometer. The thin film was cleaned by acetone, ispropanol and nitrogen, followed by spin coating ZEP520A to form a 120-nm-thick layer and baking for 1 min at 180 °C. A layer of gold with a thickness of 15 nm was sputtered on top of ZEP520A, acting as the conductive layer for electron beam lithography (EBL). The pattern was written using an Elionix ELS-7500 EX EBL system and then was developed in ZED-N50 developer for 1 min after wet etching away the gold layer. The pattern was transferred to the As_2_S_3_ layer by ICP etching (Oxford Cobra ICP Etcher). Finally, the ZEP520A mask was removed with 1165 Stripper (NMP) and a layer of PMMA A11 (MicroChem) was spin-coated onto the chip to form the Sandwich structure. The SEM image of the As_2_S_3_ pattern was captured using an Apreo S system (ThermoFisher Scientific).

### 4.2 Numerical simulation

The commercial Finite Element Method simulation package Comsol Multiphysics 5.6 was used. The measured refractive index of As_2_S_3_ from the ellipsometer was used in the simulations. A dispersionless refractive index of 1.45 was assumed for both glass substrate and PMMA superstrate. Nonlinear simulations were performed with the same numerical tool using a fully coupled solver and using values for *χ* ^(3)^(*ω*) and *χ* ^(3)^(3*ω*) extracted by using the numerical method described in Ref. [27]. Nonlinear susceptibility data was adjusted as a fit parameter to obtain best agreement between simulations and experimental results, which is legitimate because it may vary even with subtle differences in As_2_S_3_ properties.

### 4.3 Linear measurements

A home-built system consisting of a stabilized fiber-coupled light (SLS201, Thorlabs) as a source and a wide range optical spectrum analyzer (OSA) (AQ6374, Yokogawa) was setup for transmittance measurements. A polarizer (Thorlabs) was used to ensure proper polarization of the incident beam. The transmission spectrum was calculated by dividing the power transmitted through the metasurface by the power transmitted through a pure glass substrate.

### 4.4 Nonlinear measurements

A 800 nm-wavelength beam was generated from a Ti:sapphire laser with a repetition rate of 1 kHz and 100 fs output pulse width (Coherent Libra system) and split into two ultrafast optical parametric amplifier (TOPAS-C), which can each covert the center wavelength to a range from 260 to 2600 nm. The mechanical delay line stage is a Newport DL-225 with 75 nm as the minimum step. Two pulsed lasers met at a beam splitter (BP145B3, Thorlab) and were split into two branches. One branch went to a BBO crystal and the other was focused onto sample by a 20× Mitutoyo Plan Apo NIR Infinity Corrected Objective. The transmitted FF and TH were collected with an 50× Mitutoyo Plan Apo Infinity Corrected Long WD Objective with NA of 0.55. The OSA was used to measure the resonance shift with change in power intensity. When measuring TH, the transmitted light is attenuated by a NIR Absorptive ND Filter (NENIR40, Thorlabs) and focused by an N-BK7 lens with a focal length of 250 mm. Next, the FF was filtered by a short pass filter and the TH signal was coupled into a multimode fiber with an 0.50 NA, Ø400 µm core (FP400URT) connected to a Super Gamut UV-VIS-NIR Spectrometer (BaySpec, Inc). The collecting time of the spectrometer can be set from 1 to 60 s. A manual filter wheel mount with neutral density filters was used to change the power of the laser beams.

## Supplementary Material

Supplementary Material Details
