# Supplementary material for: All-optical tunable wavelength conversion in opaque nonlinear nanostructures
Source: Nanophotonics. 2022 May 30;11(17):4027–35. doi: 10.1515/nanoph-2022-0078 (PMC11501148; doi:10.1515/nanoph-2022-0078)
Supplement: Supplementary file 1 — Supplementary Material Details [file j_nanoph-2022-0078_suppl.docx]

Jiannan Gao,^1^ Maria Antonietta Vincenti,^2^ Jesse Frantz,^3^ Anthony Clabeau,^4^ Xingdu Qiao,^5^ Liang Feng,^6^ Michael Scalora,^7^ Natalia M. Litchinitser^1*^

^1Department of Electrical and Computer Engineering, Duke University, Durham, NC, 27708, USA^

^2Department of Information Engineering – University of Brescia, Via Branze 38, 25123 Brescia, Italy^

^3US Naval Research Laboratory, 4555 Overlook Ave., SW, Washington, DC 20375, USA^

^4University Research Foundation, 6411 Ivy Ln. 110, Greenbelt, MD 20770, USA^

^5Department of Electrical and Systems Engineering, University of Pennsylvania, Philadelphia, PA 19104, USA^

^6Department of Materials Science and Engineering, University of Pennsylvania, Philadelphia, PA 19104, USA^

^7Aviation and Missile Center, US Army CCDC, Redstone Arsenal, AL 35898-5000 USA^

^*natalia.litchinitser@duke.edu^

Supplementary Information for: All-optical Tunable Wavelength Conversion in Opaque Nonlinear Nanostructures

1.
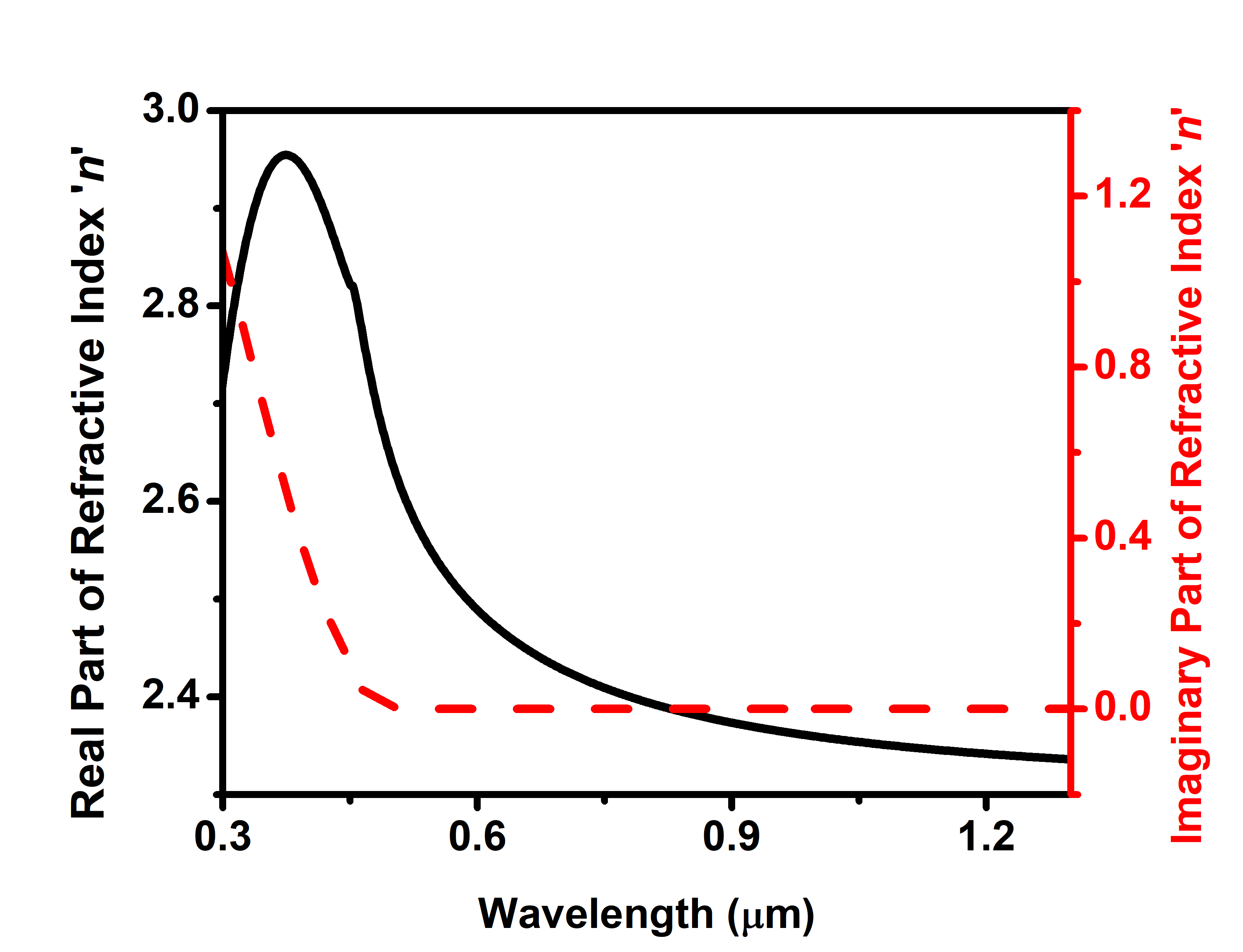
The refractive index of 300 nm As_2_S_3_ film

**Figure S1. The refractive index of 300 nm As_2_S_3_ film measured by spectroscopic ellipsometer.** It clearly indicates that As_2_S_3_ is highly dispersive and strongly absorbing at wavelengths below 500 nm, where our third harmonic wavelength (376 nm) is generated.

1.
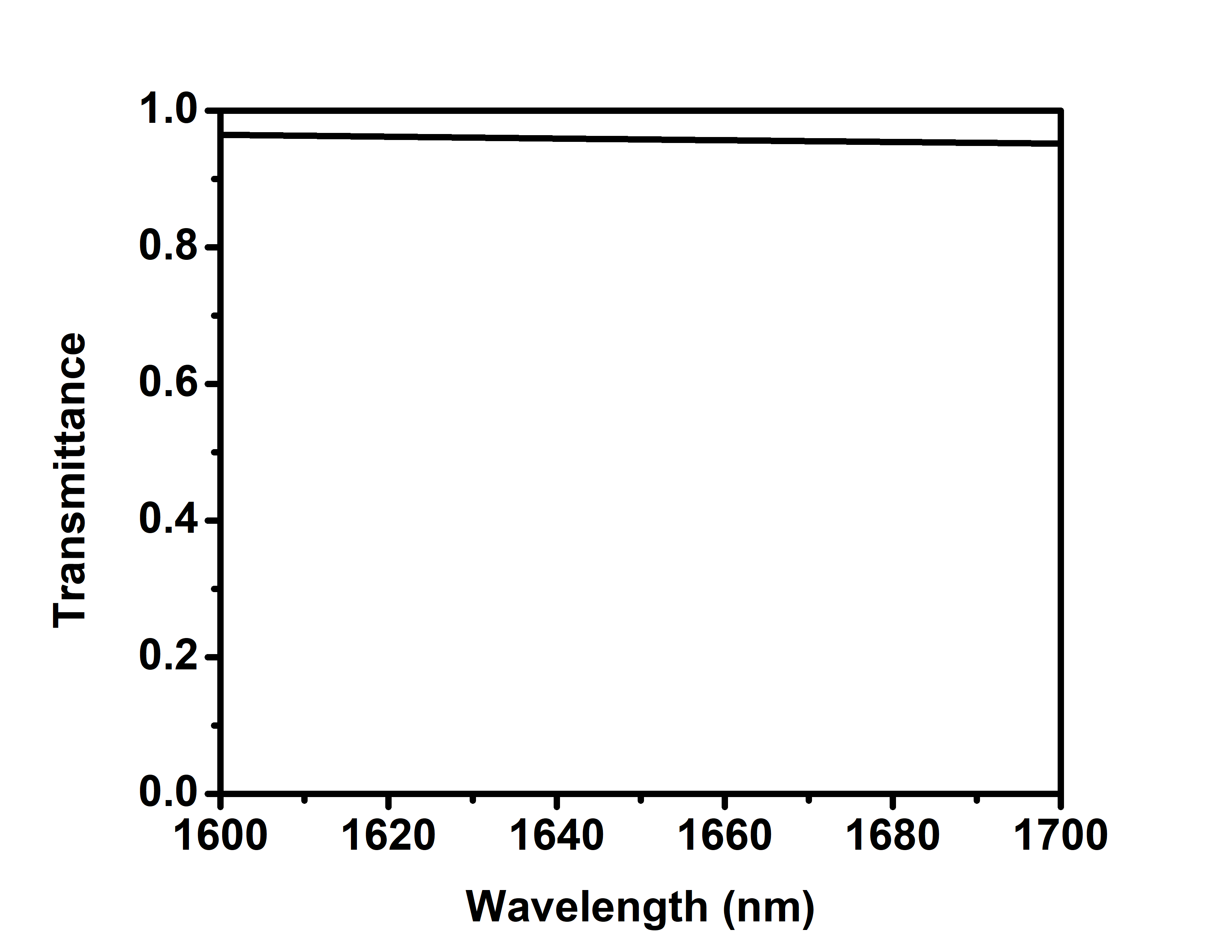
The simulated transmittance of the sample at near 1650 nm

**Figure S2.** The simulated transmittance of the sample at near 1650 nm, the wavelength of our pump beam away from any resonance.

1.
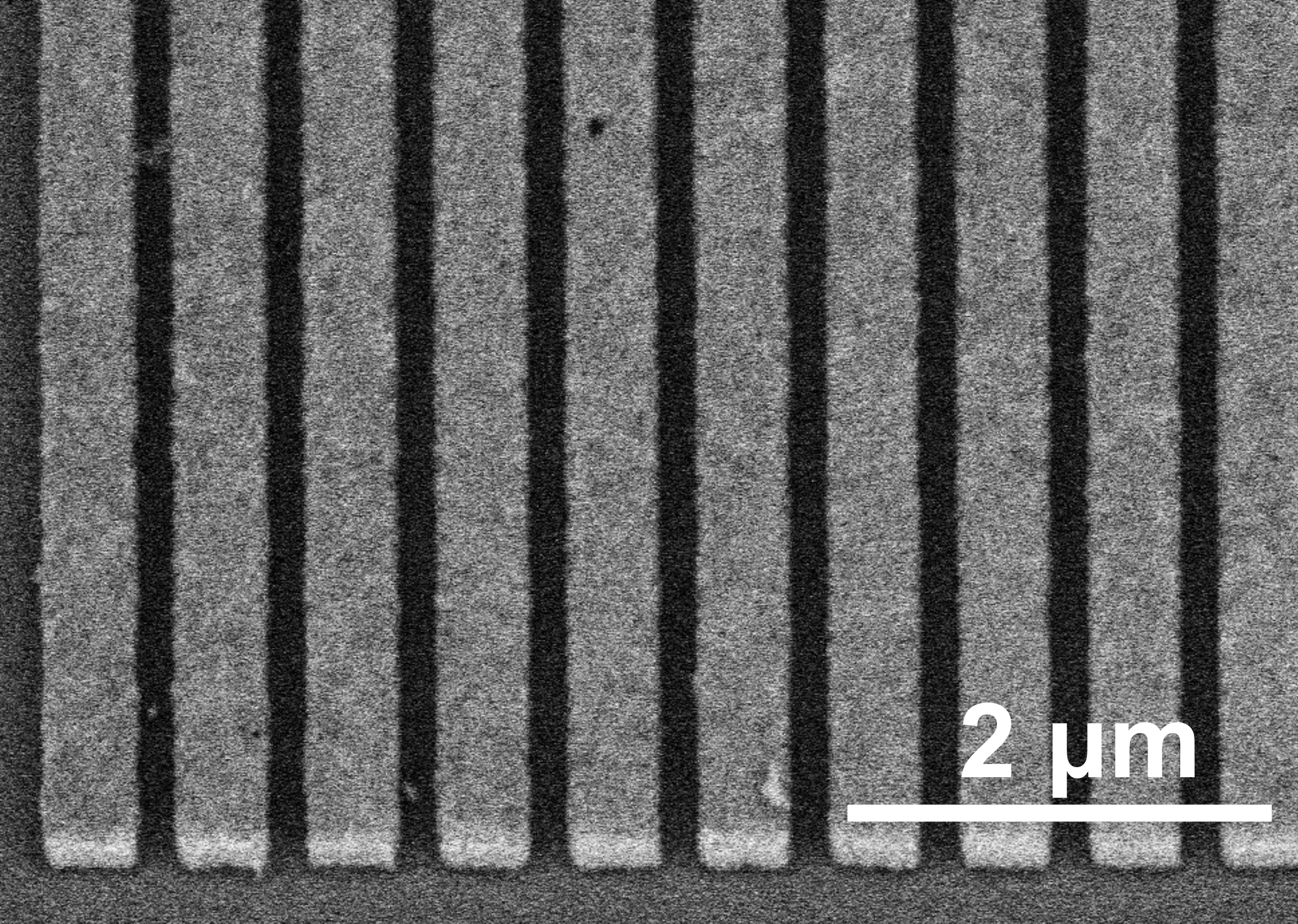
The 30-tilted scanning electron microscopy (SEM) image of the As_2_S_3_ pattern.

**Figure S3.** The 30-tilted scanning electron microscopy (SEM) image of the As_2_S_3_ pattern.

1.
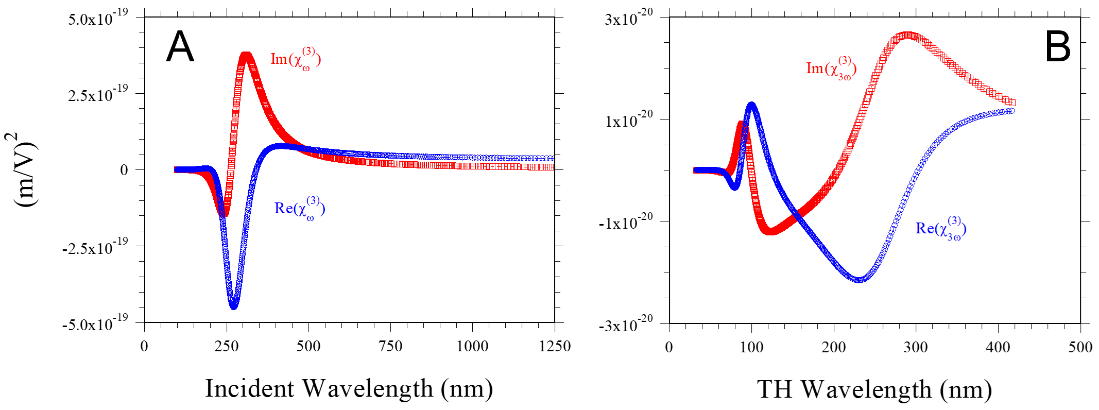
The nonlinear dispersion derived with hydrodynamic model

Figure S4. **The nonlinear dispersion derived *with* hydrodynamic model. A.** The nonlinear dispersion of fundamental wavelength. **B.** The nonlinear dispersion of TH.
